# Gingival fibroblasts produce paracrine signals that affect osteoclastogenesis in vitro

**DOI:** 10.1016/j.bonr.2024.101798

**Published:** 2024-08-20

**Authors:** Solen Novello, Ton Schoenmaker, Teun J. de Vries, Behrouz Zandieh Doulabi, Astrid D. Bakker, Marja L. Laine, Ineke D.C. Jansen

**Affiliations:** a UF Parodontologie, Pôle d'Odontologie, Centre Hospitalier Universitaire de Rennes, 35000 Rennes, France; b Unité de Formation et de Recherche d'Odontologie, Université de Rennes, 35000 Rennes, France; c Department of Periodontology, Academic Centre for Dentistry Amsterdam (ACTA), University of Amsterdam and Vrije Universiteit Amsterdam, Amsterdam, the Netherlands; d Department of Oral Cell Biology, Academic Centre for Dentistry Amsterdam (ACTA), University of Amsterdam and Vrije Universiteit Amsterdam, Amsterdam, the Netherlands

**Keywords:** Gingival fibroblasts, Peripheral blood mononuclear cells, Osteoclastogenesis, Paracrine communication, Inflammation, Periodontitis

## Abstract

In periodontitis, gingival fibroblasts (GF) appear to produce a multitude of paracrine factors. However, the influence of GF-derived soluble factors on osteoclastogenesis remains unclear. In this case study, production of paracrine factors by GF was assessed under inflammatory and non-inflammatory conditions, as well as their effect on osteoclastogenesis. Human primary GF were cultured in a transwell system and primed with a cocktail of IL-1β, IL-6 and TNF-α to mimic inflammation. GF were co-cultured directly and indirectly with human peripheral blood mononuclear cells (PBMC). Cytokines and chemokines in supernatants (flow cytometry based multiplex assay), osteoclastogenesis (TRAcP staining) and gene expression (qPCR) were quantified on days 7 and 21.

Results from this case study showed that GF communicated via soluble factors with PBMC resulting in a two-fold induction of osteoclasts. Reversely, PBMC induced gene expression of IL-6, OPG and MCP-1 by GF. Remarkably, after priming of GF with cytokines, this communication was impaired and resulted in fewer osteoclasts. This could be partly explained by an increase in IL-10 expression and a decrease in MCP-1 expression. Intriguingly, the short priming of GF resulted in significantly higher expression of inflammatory cytokines that was sustained at both 7 and 21 days.

GF appear to produce paracrine factors capable of stimulating osteoclastogenesis in the absence of physical cell-cell interactions. GF cultured in the presence of PBMC or osteoclasts had a remarkably inflammatory phenotype. Given profound expression of both pro- and anti-inflammatory cytokines after the inflammatory stimulus, it is probably the effector hierarchy that leads to fewer osteoclasts.

## Highlights

• Communication between gingival fibroblasts and PBMC is bidirectional.
• Soluble factors from gingival fibroblasts stimulate osteoclast formation.
• Osteoclast number is increased by gingival fibroblasts in indirect co-cultures.
• Osteoclast number is reduced by inflamed gingival fibroblasts in indirect co-cultures.
• PBMC induce gingival fibroblasts to express IL-6, OPG and MCP-1.

## 1 Introduction

Periodontitis is a highly prevalent multifactorial disease of the periodontium characterized by a bacterially induced chronic inflammation that may result in the progressive and irreversible destruction of the tooth supportive tissues, including gingiva, periodontal ligament, cementum and alveolar bone (Pihlstrom et al., 2005; Tonetti et al., 2017). A dysbiotic microbiome is involved in the pathogenesis of inflammatory responses in periodontitis (Hajishengallis, 2015). Several bacterial virulence factors will stimulate resident cells in the gingiva, leading to periodontal breakdown and aberrant inflammation in the periodontium (Heo et al., 2023). This complex process involves various cell-types, including gingival fibroblasts (GF), periodontal ligament fibroblasts, osteoblasts and osteoclasts. GF and periodontal ligament fibroblasts facilitate the inflammatory cascade in periodontitis (Takashiba et al., 2003), producing pro-inflammatory cytokines, like IL-1β, IL-6 and TNF-α (Naruishi, 2022; Xiong et al., 2019). These cytokines promote inflammation, osteoclast differentiation and activity in bone-degenerative diseases such as periodontitis (Boström and Lundberg, 2013). Although an important function of GF is to produce extracellular matrix and to maintain structural integrity of tissues, they also secrete numerous molecules essential for the development and function of other cell types (Costa-Rodrigues and Fernandes, 2011). They play a role in the attachment, survival and proliferation of immune cells (Moonen et al., 2018). GF also express a variety of molecules known to be involved in osteoclastogenesis, including macrophage colony-stimulating factor (M-CSF) and low levels of receptor activator of nuclear factor kappa-B ligand (RANKL) (Costa-Rodrigues and Fernandes, 2011).

Inflammatory conditions frequently give rise to osteolysis due to local enhancement of osteoclast formation and osteoclast activity (Sjöström et al., 2000). Osteoclasts are cells specialized in bone resorption, which descend from the monocyte/macrophage lineage (Boyle et al., 2003). They are multinucleated cells formed by fusion of their mononuclear precursors and subsequent differentiation (Costa-Rodrigues et al., 2010). Osteoclast formation and activation are strictly regulated by physical, autocrine and paracrine cell communication (Costa-Rodrigues and Fernandes, 2011). Under inflammatory conditions, pro-inflammatory cytokines stimulate osteoblasts and stromal cells to secrete M-CSF and RANKL (Heo et al., 2021). Chemokines also provide key signals for bone cell trafficking, differentiation and activity, playing an important role in bone remodeling and inflammation (Andrade et al., 2012). They are a large family of chemotactic cytokines produced by a number of cell types in the periodontium, such as fibroblasts, endothelial cells, macrophages, osteoclasts or monocytes (Graves, 2008). They are detected in periodontal tissues and in gingival crevicular fluid following prolonged inflammation, increasing the infiltration of inflammatory cells (Niwa et al., 2013).

However, it is unknown to what extend GF affect osteoclastogenesis in the presence and absence of inflammatory factors. In particular, a possible paracrine role of GF is still obscure.

Osteoclastogenesis induced by GF can be assessed in cell culture by adding peripheral blood mononuclear cells (PBMC) to a subconfluent layer of these fibroblasts. The GF then provide the necessary signals for the differentiation of peripheral blood monocytes to multinucleated osteoclasts (de Vries et al., 2006). Although the role of signals provided by direct cell-cell contact in this process is acknowledged, little is known about the influence of GF-derived soluble factors on osteoclastogenesis (Bloemen et al., 2010). The current study aims to identify paracrine signals produced by GF that could be involved in the formation and activation of osteoclasts in the periodontal microenvironment. In an attempt to identify paracrine factors involved, gene and protein expression of cytokines known to stimulate osteoclastogenesis were assessed: IL-1β and TNF-α (Cao et al., 2016; Cao et al., 2017). Inhibitory cytokines assessed were IL-4 (Ujiie et al., 2016) and IL-10 (Park-Min et al., 1950). IL-6 is a cytokine known to be both stimulatory and inhibitory (Palmqvist et al., 1950; Yoshitake et al., 2008). M-CSF and OPG, two key players in the osteoclastogenic process were also investigated (Costa-Rodrigues and Fernandes, 2011; Datta et al., 2008). Finally, among members of the chemokine family, MCP-1 (CCL2) and RANTES (CCL5) have been identified as typical osteoclastogenesis-associated chemokines. They play roles in mechanisms underlying osteoclast recruitment and activation (Goto et al., 2011; Brylka and Schinke, 2019).

## 2 Materials and methods

### 2.1 Gingival fibroblasts

GF were obtained from five healthy donors (three males – age 17, 17 and 30 years; 2 females – age 20 and 45 years) who underwent extraction of a third molar. The study was conducted in accordance with the Declaration of Helsinki and the protocol was approved by the Ethical Review Board of the VU Medical Center, Amsterdam (now Amsterdam UMC), the Netherlands (number 2016/105). Signed informed consent was obtained from all individuals. Periodontal tissues around the molars showed no overt signs of gingival inflammation or periodontitis (no plaque, periodontal probing ≤3 mm, no bleeding on probing, and no sign of loss of attachment).

Free gingiva and part of the intradental gingiva was cut off the tooth by means of a scalpel-knife and chopped into fragments of approximately 1 mm. The tissue fragments were washed twice in DMEM (Gibco BRL, Paisley, Scotland) supplemented with 10 % FCS (HyClone, Logan, UT), and 1 % antibiotics (100 U/ml penicillin, 100 mg/ml streptomycin, and 250 ng/ml amphotericinB [Antibiotic antimycotic solution, Sigma, St. Louis, MO]). The biopsies were cut into small pieces and divided in a 6-well dish with 1.5 ml DMEM +10 % FCS + 1 % antibiotics. The 6-well dishes were stored in a humidified atmosphere of 5 % CO2 in air at 37 °C. GF were expanded for three passages and aliquots were stored in liquid nitrogen. All experiments were performed at the 7th passage.

### 2.2 PBMC isolation

PBMC were isolated from the buffy coat of an anonymous healthy donor (blood bank, Sanquin, Amsterdam, The Netherlands) (ethical committee number NVT230.01). The buffy coat was diluted 1:1 in PBS containing 1 % citrate buffer. Twenty-five milliliter of diluted blood was carefully layered on 15 ml lymphoprep (Axisshield Po CAS, Oslo, Norway) and centrifuged for 30 min at 1200*g* without brake. The interphase containing PBMC was washed two times in PBS + 1 % citrate buffer and finally recovered in αMEM +10 % FCS + 1 % antibiotics (complete medium).

### 2.3 Co-culture experiments

Co-culture experiments were performed in 24-well plates, at 37 °C in a humidified atmosphere with 5 % CO_2_. Complete medium αMEM supplemented with 10 ng/ml human recombinant M-CSF (R&D systems, Oxon, UK) and 5 ng/ml recombinant RANKL (R&D systems, Minneapolis, MN, USA) was used for the experiment (growth medium). Culture medium was refreshed twice a week. Direct and indirect co-cultures were used.

The five cell cultures of GF were seeded one day in advance at a density of 3 × 10^4^ cells/well in 1 ml (1.58 × 10^4^ cells/cm^2^) for direct co-culture and 3 × 10^4^ cells/insert in 0.2 ml (1 × 10^5^ cells/cm^2^) for indirect co-culture. Two conditions were used: non-inflammatory conditions (complete medium) and inflammatory conditions. In the inflammatory conditions, the GF were pre-treated for 24 h with a mixture of human recombinant cytokines in complete medium to mimic inflammation: IL-1β (10 ng/ml; R&D systems), IL-6 (10 ng/ml; R&D systems), and TNFα (10 ng/ml; R&D systems).

#### 2.3.1 Direct co-culture

**Fig. 1:**
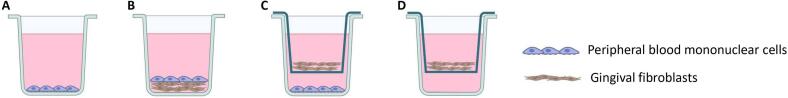
Experimental set-up. (A) Peripheral blood mononuclear cells (PBMC) in monocultures. (B) PBMC and gingival fibroblasts (GF) in direct co-cultures. (C) PBMC and GF in indirect co-cultures using inserts. (D) GF in monocultures, seeded in inserts.

At day 0, GF were washed once with PBS. PBMC in growth medium (complete medium + M-CSF and RANKL) were added to the GF cultures, at a concentration of 1 × 10^6^ cells/well in 1 ml (5.3 × 10^5^ cells/cm^2^), for 21 days (Fig. 1B).

#### 2.3.2 Indirect co-culture

Indirect co-cultures were established using cell culture inserts (Thincert, Greiner Bio-One, Kremsmünster, Austria) with 0.4 μm pore-size filter membranes. GF were seeded in the inserts, whereas PBMC were seeded on the bottom of the cell culture plates (Fig. 1C). At day 0, GF were washed with PBS. PBMC were seeded at a density of 1 × 10^6^ cells/well (5.3 × 10^5^ cells/cm^2^), for 21 days. Growth medium (complete medium + M-CSF and RANKL) was added to cover both cell layers (0.8 ml per well and 0.2 ml per insert).

As controls, GF were cultured in the upper compartment of the insert plates without PBMC underneath (Fig. 1D) and PBMC were cultured alone in wells. For PBMC alone, a quadruplicate plating was used (Fig. 1A).

### 2.4 TRAcP staining

Osteoclast quantification was performed after 21 days of culturing. Cells were fixed in 4 % PBS-buffered formaldehyde and stained for the presence of Tartrate-resistant acid phosphatase (TRAcP) using the Acid Phosphatase Leukocyte kit (Sigma-Aldrich), according to the instructions of the manufacturer. Nuclei were stained with diamidino-2-phenylindole dihydrochloride (DAPI). Micrographs were taken from ten fixed positions per well with a digital camera (Leica DFC320, Leica Microsystems, Wetzlar, Germany) and analyzed for the number of TRAcP-positive multinucleated cells. Multinucleated TRAcP+ cells with three or more nuclei were considered osteoclasts. Each condition was performed in duplicate and GF of five different donors were used. The mean of duplicate wells was used for analysis.

### 2.5 RNA analysis and real-time quantitative PCR

Quantitative polymerase chain reaction (qPCR) analysis was performed on days 7 and 21 of culture. At these time points the culture medium was removed and 300 μl of RNA lysis buffer (Qiagen, Hilden, Germany) was added per well and 200 μl per insert. Subsequently, the plates were stored in −80 °C until further use. RNA isolation was performed using the RNeasy Mini Kit (Qiagen) according to the manufacturer's instructions. The RNA concentration and quality were determined using absorption measurements at 260 and 280 nm with Synergy HT spectrophotometer (BioTek Instruments Inc., Winooski, VT, United States). RNA was reverse transcribed to cDNA using the MBI Fermentas cDNA Synthesis Kit (Vilnius, Lithuania) following the manufacturer's instructions, using both the Oligo(dT)18 and D(N)6 primers. Q-PCR primers were designed using Primer Express software, version 2.0 (Applied Biosystems, Foster City, CA, USA). To avoid amplification of genomic DNA, each amplicon spanned at least one intron. Real-time PCR was performed on the LC480 Light Cycler (Roche, Basel, Switzerland). 3 ng cDNA was used in a total volume of 10 μl containing Light Cycler SybrGreen1 Master mix (Roche) and 1 μM of each primer. Porphobilinogen deaminase (PBGD) was used as a housekeeping gene. Expression of this gene was not affected by the experimental conditions. The primer sequences used for analysis are listed in Table 1. Expression of the genes was normalized for PBGD expression following the comparative threshold (Ct) method. ΔCt (C_t gene of interest_ - C_t PBGD_) was calculated and relative expression of the genes was determined as 2^−(ΔCt)^.

**Table 1:** Primer sequences used for quantitative polymerase chain reaction (qPCR).

Gene	Sequence 5′-3′	Amplicon length (bp)	Ensemble Gene ID
*PBGD*	F: TgCAgTTTgAAATCATTgCTATgTC R: AACAggCTTTTCTCTCCAATCTTAgA	84	ENSG00000256269
*IL-1β*	F: CTTTgAAgCTgATggCCCTAAA R: AgTggTggTCggAgATTCgT	100	ENSG00000125538
*IL-4*	F: CCTggCgggCTTgAATT R: TTgAATATTTCTCTCTCATgATCgTCTT	100	ENSG00000113520
*IL-6*	F: ggCACTggCAgAAAACAACC R: ggCAAgTCTCCTCATTgAATCC	85	ENSG00000136244
*IL-10*	F: TgCCTAACATgCTTCgAgATCTC R: CAgCTgATCCTTCATTTgAAAgAA	73	ENSG00000136634
*TNF-α*	F: CCCAgggACCTCTCTCTAATCA R: gCTTgAgggTTTgCTACAACATg	103	ENSG00000111956
*DC-STAMP*	F: ATTTTCTCAgTgAgCAAgCAgTTTC R: AgAATCATggATAATATCTTgAgTTCCTT	101	ENSG00000164935
*TRAcP*	F: CACAATCTgCAgTACCTgCAAgAT R: CCCATAgTggAAgCgCAgATA	128	ENSG00000102575
*CFS1*	F: CCgAggAggTgTCggAgTAC R: AATTTggCACgAggTCTCCAT	100	ENSG00000184371
*TNFRSF11B*	F: CTgCgCgCTCgTgTTTC R: ACAgCTgATgAgAggTTTCTTCgT	100	ENSG00000164761
*MCP-1*	F: CAgCCAgATgCAATCAATgC R: TgCTgCTggTgATTCTTCTATAgCT	102	ENSG00000108691
*RANTES*	F: CATCTgCCTCCCCATATTCCT R: TgCCACTggTgTAgAAATACTCCTT	104	ENSG00000161570

PBGD, porphobilinogen deaminase; IL-1 beta, interleukine 1β; IL-4, interleukine 4; IL-6, interleukine 6; IL-10, interleukine 10; TNF-α, tumor necrosis factor-alpha; DC-STAMP, dendritic cell-specific transmembrane protein; TRAcP, tartrate-resistant acid phosphatase; CFS1, colony-stimulating factor1 (coding for macrophage-colony stimulating factor (M-CSF)); TNFRSF11B, tumor necrosis factor receptor superfamily member 11b, (coding for osteoprotegerin (OPG)); MCP-1, monocyte chemoattractant protein-1; RANTES, regulated on activation, normal T cell expressed and secreted.

### 2.6 LEGENDplex cytokines and chemokines profiling analysis

To analyze protein concentrations of IL-1β, IL-4, IL-6, IL-10, TNF-α, OPG, MCP-1(CCL2) and RANTES (CCL5) in the conditioned medium collected after seven days from monocultures and co-cultures, a custom designed LEGENDplex Multi-Analyte Flow Assay Kit (BioLegend, San Diego, CA, USA) was used according to the manufacturer's instructions.

Data were analyzed using BD Accuri C6 Flow Cytometer (BD Biosciences, East Ruthford, NJ, USA). Final analysis of the data was performed using LEGENDplex online software (https://legendplex.qognit.com). Protein concentrations were calculated using the generated standard curve and expressed in picograms per milliliter (pg/ml).

### 2.7 Statistical analysis

All data were analyzed using GraphPad Prism software version 8.1.0 (GraphPad Software, San Diego, CA, United States). Initially, normality was tested using the Shapiro–Wilk test. One-way ANOVA with a Tukey's multiple comparisons test (normally distributed data) or Kruskal Wallis with Dunn's multiple comparisons test if not normally distributed was used to compare three or more groups. A paired *t*-test (normally distributed data) or a Wilcoxon signed-rank test (non-normally distributed data) was used to compare two groups. Values were considered to be significantly different when *p* ≤ 0.05.

## 3 Results

### 3.1 GF communicate with osteoclasts precursors

#### 3.1.1 GF stimulate osteoclast formation in a paracrine way

**Fig. 2:**
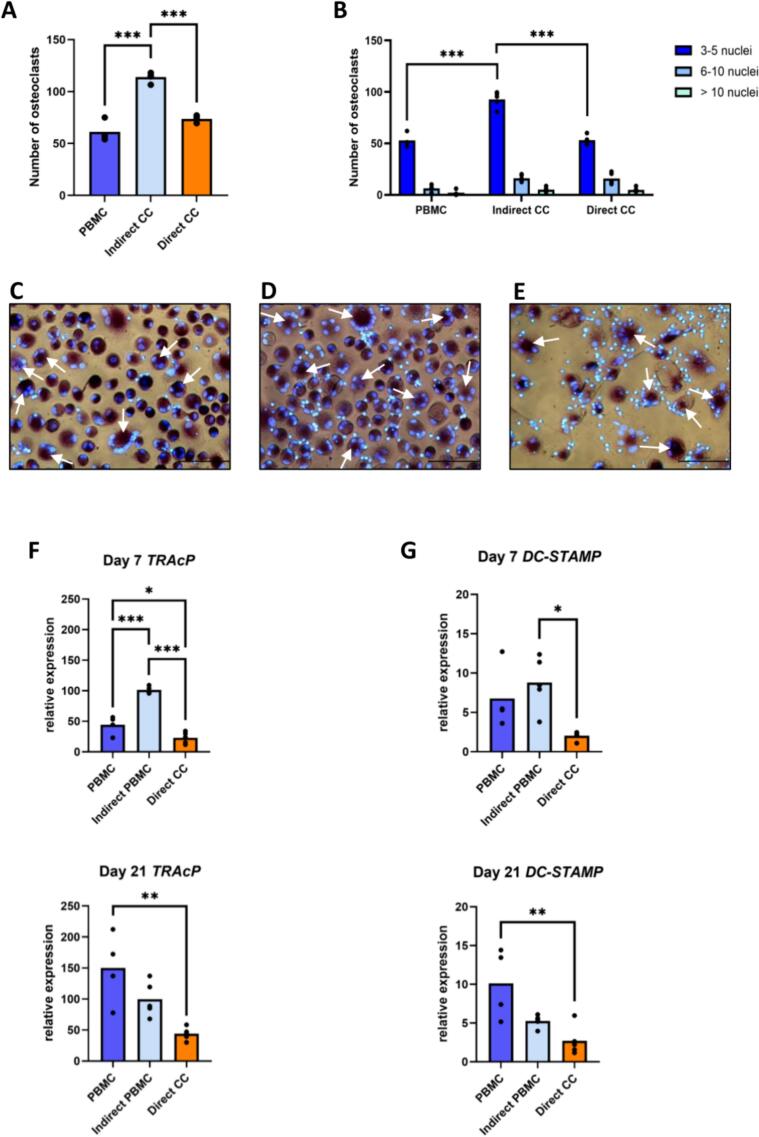
GF stimulate osteoclast formation in a paracrine way. (A) Quantification of the number of TRAcP+ cells per 10 standardized pictures per well, after 21 days of culture (PBMC), indirect co-culture with GF (Indirect CC) or direct co-culture with GF (Direct CC). (B) Number of nuclei per TRAcP+ cells in monocultures and co-cultures, after 21 days. (C, D, E) Micrographs of PBMC after 21 days of culture. Cells were stained for TRAcP activity (purple) and nuclei were stained with 4′,6-diamidino-2-fenylindool (blue). TRAcP+ multinucleated cells were considered to be osteoclasts with at least three nuclei. Some osteoclasts are depicted with white arrows. Magnification 20×. (C) Micrographs of PBMC after 21 days of culture. (D) Micrographs of PBMC after 21 days of indirect co-culture with GF. (E) Micrographs of PBMC after 21 days of direct co-culture with GF. (F, G) Relative gene expression of osteoclast genes TRAcP (F) and DC-STAMP (G) by PBMC in monoculture (PBMC), in indirect co-culture (Indirect PBMC) and by GF + PBMC in direct co-culture (Direct CC), at day 7 and day 21. Data are presented as mean (one-way ANOVA with a Tukey's multiple comparisons post hoc test) * *p* ≤ 0.05. ** *p* ≤ 0.01. *** *p* ≤ 0.001. For PBMC alone, a quadruplicate plating was used, the co-cultures were with *n* = 5 GF donors.

In order to investigate whether GF communicate with PBMC, PBMC were either monocultured or co-cultured directly or indirectly with GF for 21 days and stained with TRAcP and DAPI to visualize TRAcP positive cells. Multinucleated TRAcP+ cells with three or more nuclei were considered osteoclasts (Fig. 2C, D, E). Two-fold more osteoclasts were formed in indirect co-culture, where GF were seeded in the transwell (*p* ≤ 0.001). Similar numbers of osteoclasts were formed in the cultures with PBMC alone and in the direct co-cultures (Fig. 2A). Regarding the number of nuclei, this augmentation was related to an increase of osteoclasts with 3 to 5 nuclei. Osteoclasts with 6 to 10 nuclei were significantly less numerous in monocultured PBMC (*p* = 0.02). No difference could be demonstrated in the group with >10 nuclei. This category of multinucleated cells was barely encountered (Fig. 2B).

Relative gene expression of two markers of osteoclast differentiation (*TRAcP and DC-STAMP*) is shown in Fig. 2F and G. At day 7, expression of both genes was significantly increased in PBMC in indirect co-culture, reflecting the number of osteoclasts (*p* ≤ 0.001 for *TRAcP* and *p* = 0.0105 for *DC-STAMP*). For both markers, expression increased between day 7 and day 21 for monocultured PBMC.

#### 3.1.2 GF modify PBMC gene expression

**Fig. 3:**
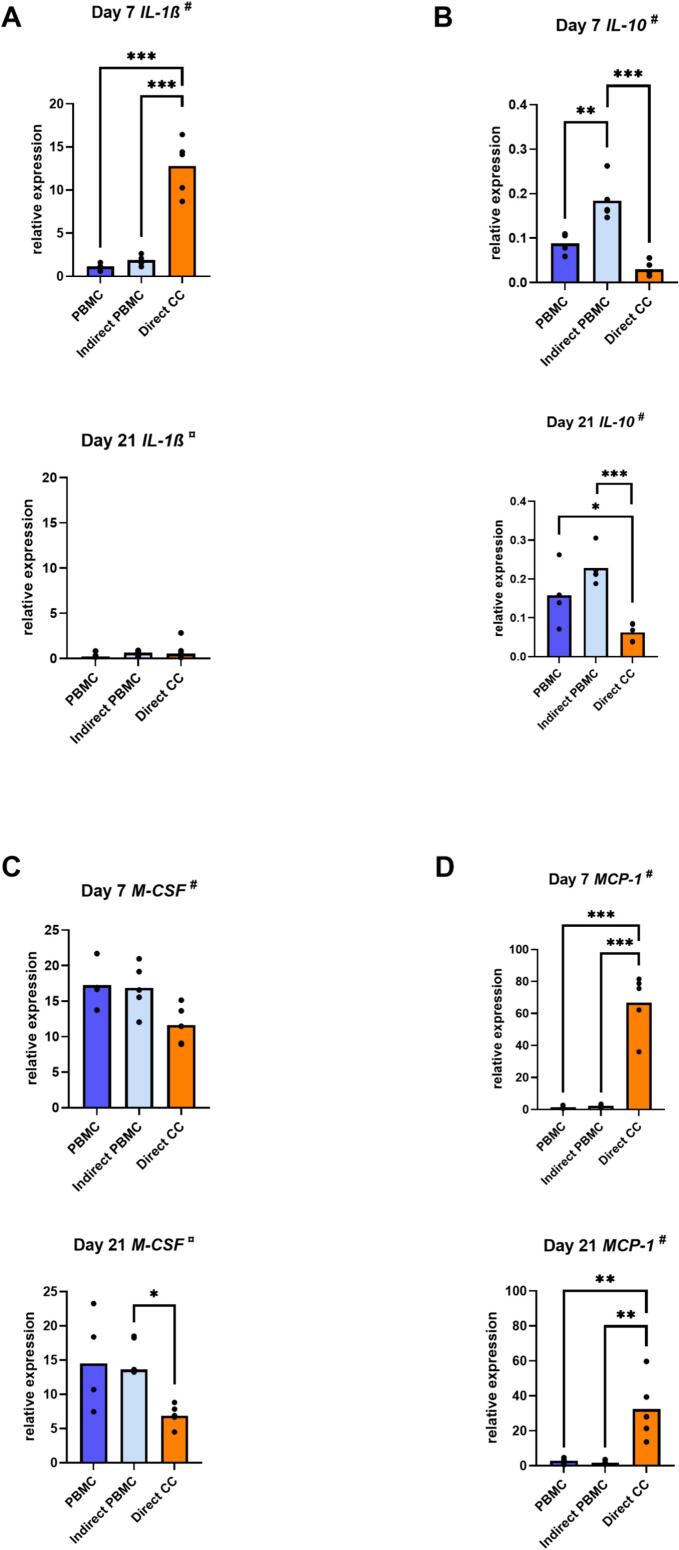
In the presence of GF, PBMC gene expression was modified. Relative gene expression of IL-1β (A), IL-10 (B), M-CSF (C) and MCP-1 (D) by PBMC in monoculture (PBMC), in indirect co-culture (Indirect PBMC) and by GF + PBMC in direct co-culture with GF (Direct CC), at day 7 and day 21. ^#^ Data are presented as mean (one-way ANOVA with a Tukey's multiple comparisons post hoc test). ¤ Data are presented as median (Kruskal Wallis with Dunn's multiple comparisons test). * *p* ≤ 0.05. ** *p* ≤ 0.01. *** *p* ≤ 0.001. For PBMC alone, a quadruplicate plating was used, the co-cultures were with *n* = 5 GF donors.

To investigate the effect of GF on PBMC gene expression, qPCR analysis was performed for several genes associated with osteoclast formation or inflammation. PBMC gene expression of the pro-inflammatory marker *IL-1β*, the anti-inflammatory marker *IL-10*, the monocyte proliferation marker *M-CSF* and the chemokine *MCP-1* showed the largest differences between the conditions and time points. In direct co-cultures, expression of *IL-1β* and *MCP-1* was significantly higher compared to the other conditions (Fig. 3A, D) and expression of *IL-10* and *M-CSF* (day 21) was significantly lower (Fig. 3B, C). Expression of *IL-10* was highest in indirect co-cultures (Fig. 3B).

Gene expression levels of *IL-6* and *OPG* by PBMC were undetectable or very low (data not shown). Co-culture did not affect *TNF-α*, *IL-4* and *RANTES* expression under any conditions (data not shown).

#### 3.1.3 GF influence protein secretion in direct and indirect co-cultures

**Fig. 4:**
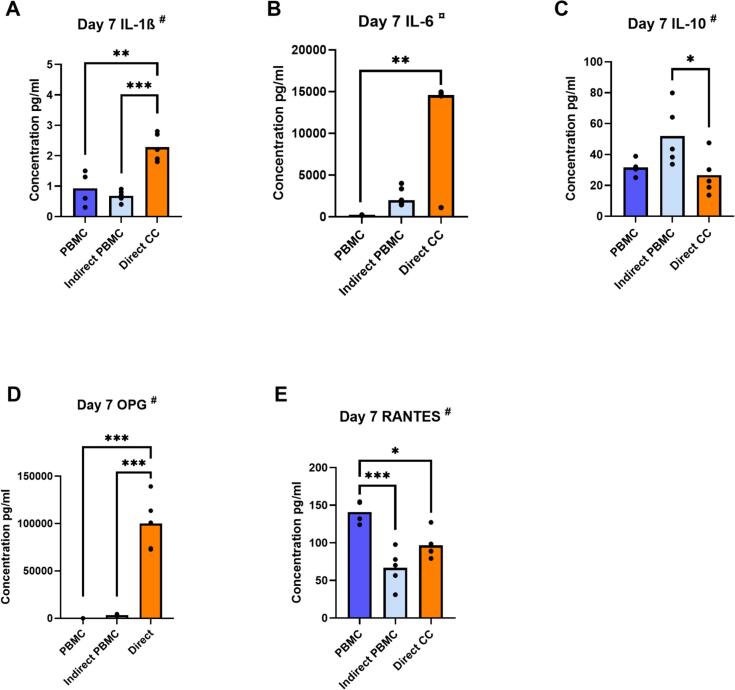
Presence of GF influence protein secretion in direct and indirect co-cultures. Protein concentrations of IL-1β (pg/ml) (A), IL-6 (pg/ml) (B), IL-10 (pg/ml) (C), OPG (pg/ml) (D) and RANTES (pg/ml) (E) in supernatants from PBMC in monoculture (PBMC), from the lower compartment in indirect co-culture (Indirect PBMC) and from GF + PBMC in direct co-culture (Direct CC) at day 7, using a LEGENDplex Multi-Analyte FlowAssay Kit. ^#^ Data are presented as mean (one-way ANOVA with a Tukey's multiple comparisons post hoc test). ¤ Data are presented as median (Kruskal Wallis with Dunn's multiple comparisons test). * *p* ≤ 0.05. ** *p* ≤ 0.01. *** *p* ≤ 0.001. For PBMC alone, a quadruplicate plating was used, the co-cultures were with *n* = 5 GF donors.

Protein concentrations in supernatants of PBMC alone or in direct and indirect co-culture with GF (lower compartment) are shown in Fig. 4. Protein concentration of IL-1 β, IL-6 and OPG increased in direct co-cultures (Fig. 4A, B, D). Conversely, protein concentration of RANTES was significantly higher in monocultures (Fig. 4E) and protein concentration of IL-10 was increased in indirect co-cultures (Fig. 4C). Protein concentration of TNF-α, IL-4 and MCP-1 was unaffected by the co-culture (data not shown).

### 3.2 In indirect co-culture, PBMC influence GF gene expression and protein secretion

The above data have shed light on the influence of GF on PBMC. The transwell system likewise allows to investigate how PBMC influence gene expression of GF.

#### 3.2.1 Presence of PBMC strongly upregulates expression of *IL-6*, *OPG* and *MCP-1* by GF

**Fig. 5:**
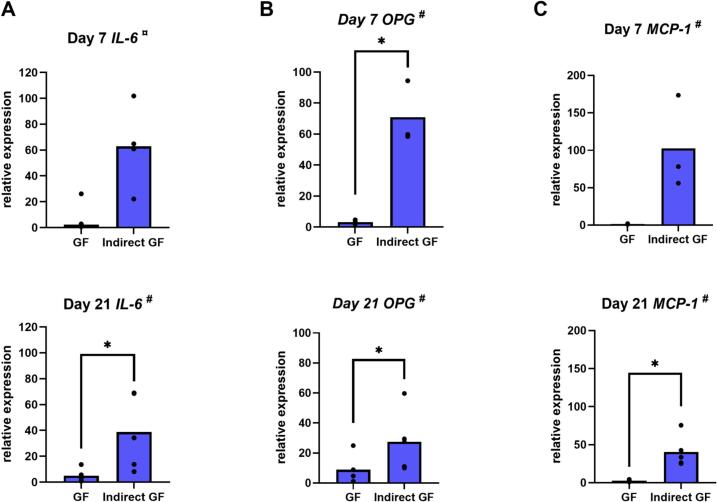
Presence of PBMC increases IL-6, OPG and MCP-1 gene expression by GF. Relative gene expression of IL-6 (A), OPG (B) and MCP-1 (C) by GF in monoculture (GF) and in indirect co-culture with PBMC (Indirect GF), at day 7 and day 21. ^#^ Data are presented as mean (paired *t*-test). ¤ Data are presented as median (Wilcoxon test). * *p* ≤ 0.05. *n* = 5 GF donors.

GF expressed more *IL-6* (*p* = 0.04), *OPG* (*p* = 0.02) and *MCP-1* (*p* = 0.01) when indirectly co-cultured with PBMC, especially at day 21 (Fig. 5). Gene expression levels of *IL-1β*, *TNF-α*, *IL-4*, *IL-10* and *RANTES* was very low or undetectable under both conditions (data not shown). Presence of PBMC had no effect on *M-CSF* expression by GF (data not shown).

#### 3.2.2 Presence of PBMC upregulates proteins associated with inflammation

**Fig. 6:**
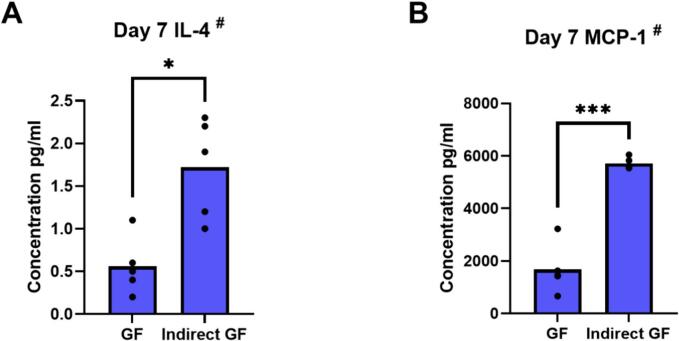
Presence of PBMC upregulates protein levels in GF supernatants. Protein concentrations of IL-4 (pg/ml) (A) and MCP-1 (pg/ml) (B) in supernatants from GF in monoculture (GF) and from the upper compartment in indirect co-culture with PBMC (Indirect GF) at day 7, using a LEGENDplex Multi-Analyte FlowAssay Kit. ^#^ Data are presented as mean (paired t-test). * p ≤ 0.05. ** *p* ≤ 0.01. *** *p* ≤ 0.001. n = 5 GF donors.

In indirect co-culture, protein concentration of IL-4 (p = 0.04) and MCP-1 (*p* ≤ 0.001), measured in the upper compartment, were increased compared to monocultures (Fig. 6A, B). Protein concentration of IL-6, IL-10, RANTES and OPG also seemed to be higher, although this was not significant (data not shown). Protein concentration of IL-1β and TNF-α was very low and unaffected by the presence of PBMC (data not shown).

### 3.3 Priming of GF with pro-inflammatory cytokines has long-lasting effects on osteoclast formation, gene expression and protein secretion

Having shown that GF communicate with PBMC, and vice versa, we next investigated whether a 24-h priming of GF with pro-inflammatory cytokines altered this communication.

#### 3.3.1 Priming of GF with inflammatory cytokines reduces osteoclast formation

**Fig. 7:**
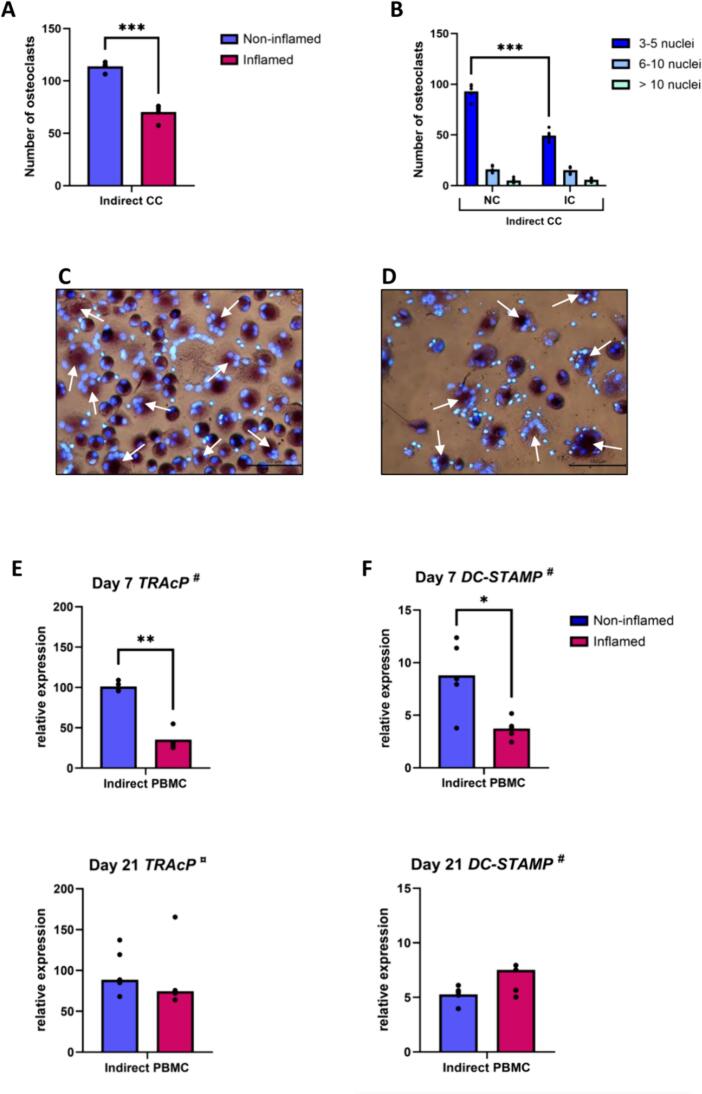
A 24-h priming of GF with pro-inflammatory cytokines decreases osteoclast formation. (A) Quantification of the number of TRAcP+ cells per 10 standardized pictures per well, after 21 days of indirect co-culture with GF pre-treated (inflamed) or not (non-inflamed) with inflammatory cytokines. Data are presented as mean (paired t-test). (B) Number of nuclei per TRAcP+ cells after 21 days of indirect co-cultures, under non-inflammatory conditions (NC) or inflammatory conditions (IC). Data are presented as mean (one-way ANOVA with a Tukey's multiple comparisons post hoc test). (C) Micrographs of PBMC after 21 days of indirect co-culture with GF. (D) Micrographs of PBMC after 21 days of indirect co-culture with GF pre-treated with inflammatory cytokines. Some osteoclasts are depicted with white arrows. Magnification 20×. (E, F) Relative gene expression of osteoclast genes TRAcP (E) and DC-STAMP (F) by PBMC in indirect co-culture with GF pre-treated (inflamed) or not (non-inflamed) with inflammatory cytokines, at day 7 and day 21. # Data are presented as mean (paired t-test). ¤ Data are presented as median (Wilcoxon test). * p ≤ 0.05. ** p ≤ 0.01. *** p ≤ 0.001. The co-cultures were with n = 5 GF donors.

In indirect co-cultures, the number of osteoclasts was significantly lower after exposure of GF to inflammatory cytokines (p ≤ 0.001) (Fig. 7A, C, D). This is linked to a reduction in the number of osteoclasts with 3 to 5 nuclei (Fig. 7B). In agreement with this decrease, relative gene expression of *TRAcP* (*p* = 0.005) and *DC-STAMP* (*p* = 0.03) was significantly lower at day 7 in the presence of treated GF. At day 21, no difference was observed between the two culture conditions (Fig. 7E, F).

#### 3.3.2 Inflammation primed GF increases PBMC gene expression of *IL-1β*, *TNF-α*, *IL-4*, *MCP-1* and *RANTES* and decreases IL-10 expression

**Fig. 8:**
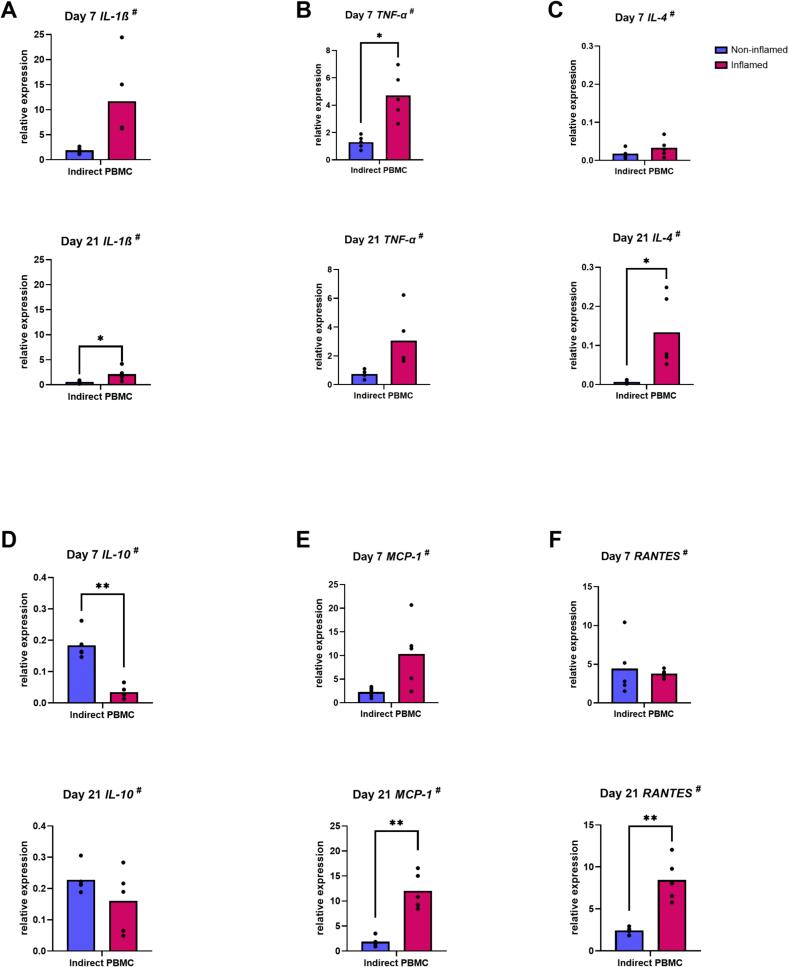
Inflammation primed GF increases PBMC gene expression of IL-1β, TNF-α, IL-4, MCP-1 and RANTES and decreases IL-10 expression. Relative gene expression of IL-1β (A), TNF-α (B), IL-4 (C), IL-10 (D), MCP-1 (E) and RANTES (F) by PBMC in indirect co-culture with GF pre-treated (inflamed) or not (non-inflamed) with inflammatory cytokines, at day 7 and day 21. ^#^ Data are presented as mean (paired *t*-test). * *p* ≤ 0.05. ** *p* ≤ 0.01. The co-cultures were with *n* = 5 GF donors.

Pre-treatment of GF with inflammatory cytokines resulted in an increased expression of *TNF-α* (*p* = 0.01) at day 7 and *IL-1β* (*p* = 0.05), *IL-4* (*p* = 0.03), *MCP-1* (*p* = 0.006) and *RANTES* (*p* = 0.005) at day 21 by PBMC in indirect co-culture (Fig. 8A, B, C, E, F) and a decreased expression of *IL-10* (*p* = 0.004) (Fig. 8D). It had no effect on the expression of *IL-6*, *M-CSF* and *OPG* by PBMC (data not shown).

#### 3.3.3 Priming of GF increases IL-1β, TNF-α and OPG protein concentration in PBMC supernatants

**Fig. 9:**
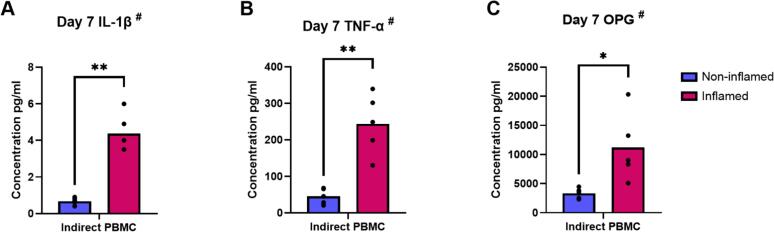
Priming of GF increases IL-1β, TNF-α and OPG protein concentration in PBMC supernatants. Protein concentrations of IL-1β (pg/ml) (A), TNF-α (pg/ml) (B) and OPG (pg/ml) (C) in supernatants from PBMC (lower compartment) after 7 days of indirect co-culture with GF pre-treated (inflamed) or not (non-inflamed) with inflammatory cytokines, using a LEGENDplex Multi-Analyte FlowAssay Kit. ^#^ Data are presented as mean (paired *t*-test). * *p* ≤ 0.05. ** *p* ≤ 0.01. The co-cultures were with *n* = 5 GF donors.

Pro-inflammatory cytokine priming of GF led to increased levels of IL-1β (*p* = 0.001), TNF-α (p = 0.006) and OPG (*p* = 0.03) proteins in the lower compartment of the transwells. (Fig. 9). No effect on protein concentration of IL-6, IL-4, IL-10, MCP-1 and RANTES was detected (data not shown).

#### 3.3.4 Priming of GF with pro-inflammatory cytokines increases their gene expression of *IL-6*

**Fig. 10:**
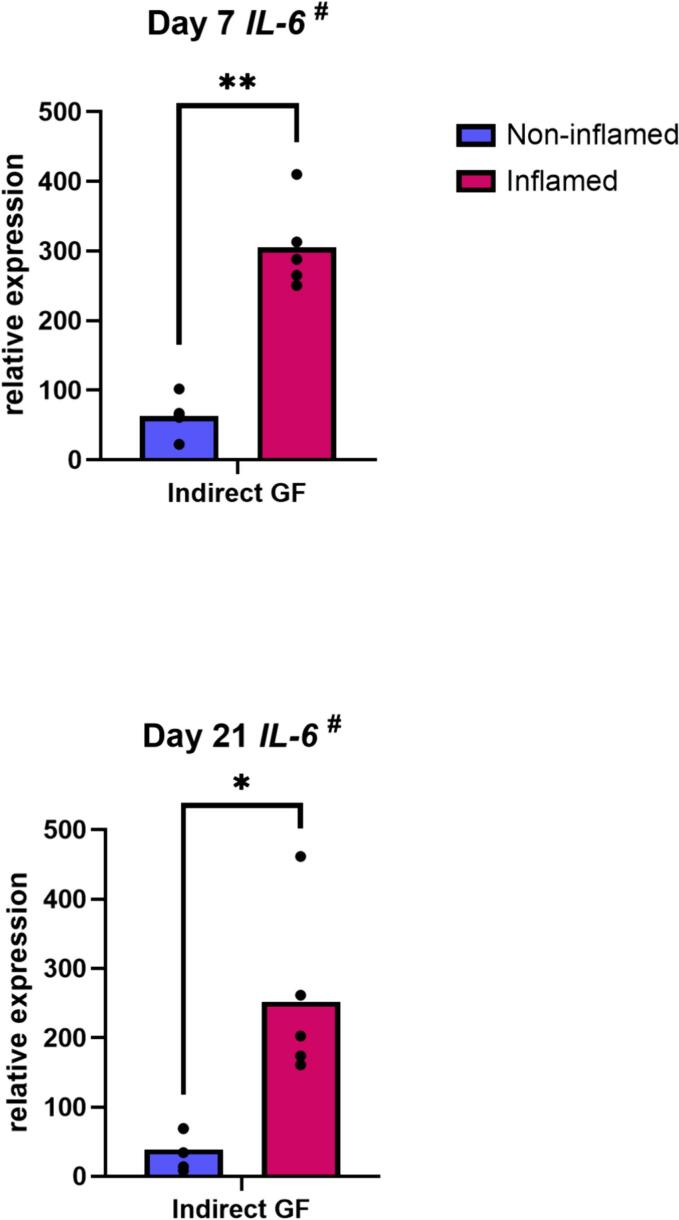
Priming of GF with pro-inflammatory cytokines increases their gene expression of IL-6. Relative gene expression of IL-6 by GF in indirect co-culture with PBMC, with (inflamed) or without (non-inflamed) pre-treatment with inflammatory cytokines, at day 7 and day 21. ^#^ Data are presented as mean (paired *t*-test). * *p* ≤ 0.05. ** *p* ≤ 0.01. *n* = 5 GF donors.

Exposure of GF to inflammatory cytokines led to an increased expression of *IL-6* (*p* = 0.002 at day 7; *p* = 0.03 at day 21) (Fig. 10). Gene expression of *TNF-α*, *IL-4*, *IL-10* and *RANTES* were undetectable or very low under all conditions. Priming did not affect *IL-1β, M-CSF*, *OPG* and *MCP-1* gene expression by GF (data not shown).

#### 3.3.5 Inflammatory pre-treatment of GF increases levels of IL-10 protein and decreases levels of IL-6 and MCP-1 proteins in GF supernatants

**Fig. 11:**
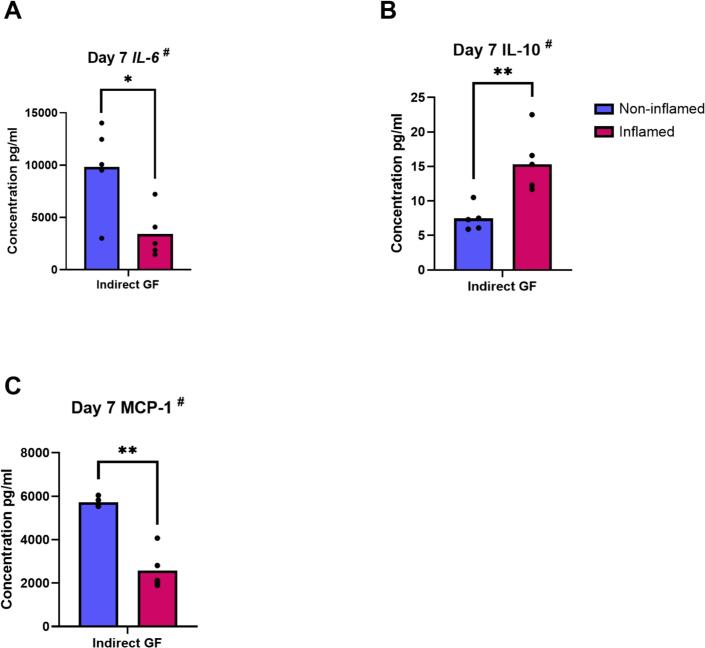
Inflammatory pre-treatment of GF increases levels of IL-10 protein and decreases levels of IL-6 and MCP-1 proteins in GF supernatants. Protein concentrations of IL-6 (pg/ml) (A), IL-10 (pg/ml) (B) and MCP-1 (pg/ml) (C) in supernatants from GF pre-treated (inflamed) or not (non-inflamed) with inflammatory cytokines, after 7 days of indirect co-culture with PBMC, using a LEGENDplex Multi-Analyte FlowAssay Kit. ^#^ Data are presented as mean (paired t-test). * p ≤ 0.05. ** p ≤ 0.01. n = 5 GF donors.

After a pulse with inflammatory cytokines, the upper compartment of the transwell contained less IL-6 (p = 0.03) and MCP-1 (p = 0.002) and more IL-10 (*p* = 0.006) (Fig. 11). Protein concentration of IL-1β and RANTES in GF supernatant appeared to increase under inflammatory conditions, although this was not significant. In a similar way, IL-4 concentration appeared to drop in the presence of inflammation (data not shown). Concentration of TNF-α and OPG was unaffected by the presence of inflammation (data not shown).

## 4 Discussion

The inflammatory process of periodontitis is accompanied by an increased release of cytokines and chemokines, leading to soft tissue destruction and bone resorption (Ramadan et al., 2020). The persistence of a chronic inflammatory immune response stimulates the formation and activity of osteoclasts, which in turn resorb alveolar bone. Osteoclastogenesis is determined as the differentiation of osteoclast precursors into multinucleated osteoclasts (Boyle et al., 2003; Jansen et al., 2012). This process requires the action of several growth factors and cellular interactions, but in particular M-CSF and RANKL are crucial and used worldwide as key molecules in osteoclastogenesis assays (Boyle et al., 2003). Hence, we have chosen to study the effect of GF in direct and indirect osteoclastogenesis in the presence of these two cytokines. In vivo, especially in inflamed tissues, fibroblasts are of great importance (Costa-Rodrigues et al., 2010). GF are the most abundant cells in gingival connective tissue and play an important role in the control of inflammation in inflamed gingiva. GF responses to elevated inflammatory cytokines are thought to contribute to the development of periodontitis (Ruppeka-Rupeika et al., 2018; Sawada et al., 2013). In the current study, we postulated that soluble factors secreted by GF could interfere with osteoclastogenesis. For this purpose, an indirect co-culture system was used. Five GF donors were used but only one PBMC donor, making this study rather a case study. Our results demonstrated that GF are able to induce osteoclastogenesis in the absence of physical cell-cell interactions. Conversely, PBMC increased the expression of some genes by GF.

After 21 days of culture, cytokine and chemokine gene expression and protein production by PBMC were affected by the presence of GF. They communicated with PBMC via soluble factors, resulting in an increased osteoclast formation. They did not contribute significantly to osteoclast formation when in direct cell-cell contact, since similar numbers of osteoclasts were found in PBMC alone and in direct co-cultures with GF. Although some studies have shown that direct cell-cell contact between osteoclasts precursors and fibroblasts is required and results in a higher number of osteoclasts (de Vries et al., 2006; Bloemen et al., 2010; Uchiyama et al., 2009), our data showed that the secretion of soluble factors could contribute to the increased formation of osteoclasts. The following experiments could assess the functionality of these osteoclast-like cells, for example by evaluating resorption pits on bone slices. Our qPCR results revealed that osteoclast precursors expressed gene associated with fusion (*DC-STAMP*) and generic osteoclast marker gene (*TRAcP*). At day 7, the expression was highest in indirect co-culture, the condition in which we found the highest number of osteoclasts with TRAcP staining. *TRAcP* and *DC-STAMP* expression was lower in direct co-culture. However, it has to be noted that direct comparison of expression levels between direct and indirect co-cultures is difficult since the RNA isolated in the direct co-cultures comes from both the GF and the PBMC, whereas the RNA isolated in the indirect co-cultures comes from the individual cell populations separately. Similarly, the protein concentration in direct co-culture supernatants comes from both PBMC and GF and in indirect co-cultures, exchanges are possible between the upper and lower compartments through the pores of the inserts. It is therefore not possible to determine whether the proteins detected originate from PBMC or GF. Although it is possible, even likely, that GF in vivo are in direct contact with monocytes, osteoclasts do not form in the gingiva. They do so on bone, at a distance from GF, where osteocytes of the alveolar bone express RANKL, probably because they need a very rigid substrate (Sprangers et al., 2016a; Graves et al., 2018). This suggests that contribution of GF to osteoclast formation should be via a paracrine communication between the two cell types. So, to get closer to the in vivo situation, we have chosen to focus on indirect communication.

Interestingly, in this case study, the communication between GF and PBMC was bidirectional. As discussed above, GF induced gene expression of PBMC /osteoclast precursors. At least as potent, PBMC influenced gene expression of GF in indirect co-cultures**.** For three genes (*IL-6*, *OPG* and *MCP-1*), we observed that when GF were in indirect co-cultures with PBMC, gene expression increased sharply. This same increase was found when the GF were monocultured with pro-inflammatory cytokines (data not shown). This evokes the invasion of monocytes to the site during the inflammatory reaction, where they release pro-inflammatory cytokines (Gonçalves et al., 2010). MCP-1 (CCL2) is a key mediator of osteoclastogenesis, well-known as a potent chemotactic factor for monocytes. It binds its receptor on osteoclast precursors to drive osteoclast differentiation (Brylka and Schinke, 2019; McDonald et al., 2021; Mulholland et al., 2019). The increase in *MCP-1* expression by GF in indirect co-culture, like that of *IL-6*, could explain greater osteoclastic induction. However, the high gene expression of *OPG* by GF in indirect co-culture without priming seems to be in contradiction with the increase in osteoclast numbers, which has already been described in other studies (de Vries et al., 2006; Bloemen et al., 2010). However, OPG was only weakly detectable in the lower compartment of the transwells. Possibly OPG is not able to pass through the membrane and therefore cannot exert its inhibitory effect on osteoclastogenesis in this indirect co-culture. It might be encapsulated in vesicles that are too large to pass the 0.4 μm membrane (Aoki et al., 2010). Also, all assays were performed in the presence of recombinant RANKL. Likewise, it is possible that osteoclast formation is not induced by RANKL, as robustly demonstrated by Kim et al. who suggest the possibility that an alternative pathway of osteoclast differentiation may exist (Kim et al., 2005). In this study, analysis of RANKL expression was equally important, but we were not able to detect it. Concentrations of two proteins studied also increased sharply in the upper compartment when GF were co-cultured with PBMC. To our knowledge, this is a new finding and sheds light on how immune cells may affect fibroblasts in periodontitis. Our results demonstrate that immune cells, even at distance, without direct cell-cell contact, can alter the inflammatory phenotype of GF. Next experiments should indicate whether this has even more consequences, such as on the level of collagen I synthesis or on the induction of proteases.

Based on previously published data, IL-1β, IL-6 and TNF-α were chosen, in order to mimic the inflammatory environment that can be found in periodontitis (Pathak et al., 2016). These cytokines appear to promote osteoclast formation and activity in the inflamed bone observed in periodontitis (Sakata et al., 1999). Increased levels of IL-1β, TNF-α and IL-6 have been detected in periodontal lesions (Caldeira et al., 2021). They play central roles in the early phase of periodontitis, surrounding GF in inflamed gingival tissue and triggering inflammatory responses (Naruishi and Nagata, 2018). In this case study, GF were pre-treated for 24 h with these three inflammatory cytokines. This time frame was found in several studies (Sawada et al., 2013; Morandini et al., 2011). Our results showed an effect of the 24-h inflammatory pre-treatment after 7 and even 21 days for some markers. Gene expression of both PBMC and GF was still affected at day 21, showing that this short activation of GF resulted in long term effects. This could help to understand the bone remodeling affected by inflammation in periodontitis.

Unexpectedly, priming GF with pro-inflammatory cytokines led to a reduction in the number of osteoclasts in indirect co-cultures comparable to the level observed in PBMC monocultures. The stimulatory effect of GF on osteoclast formation disappeared, which is also consistent with the decreased expression of *TRAcP* and *DC-STAMP* genes. (Moonen et al., 2018). Nagasawa et al. also found that the culture supernatant of LPS-stimulated GF significantly reduced the number of TRAcP positive cells generated by culturing monocytes with RANKL and M-CSF, possibly mediated by increased OPG expression (Nagasawa et al., 2002). In line with this, in this case study, this could be due to the increased OPG concentration in the lower compartment under these conditions. Another interpretation is that apoptosis might be involved. However, upon visual inspection throughout the culture, there is no reason to believe that apoptosis is at stake, especially since M-CSF and RANKL were supplied throughout the culture. Moonen et al. also previously reported that GF contribute to the maintenance of PBMC (Moonen et al., 2018).

Cytokine pre-incubation of GF also altered the gene expression of PBMC in indirect co-culture. *IL-1β* and *TNF-α* gene expression increased in the presence of treated GF, suggesting that the GF secrete soluble factors that affect PBMC gene expression in a paracrine manner. Moreover, the stimulus itself therefore seems to switch on the gene even when the added stimulus has been washed away for at least 7 days. This is in agreement with Bloemen et al. who showed that a 6-h stimulation with IL-1β resulted in an endured expression of *IL-1β* (Bloemen et al., 2011). Inflammatory mediators are usually described as positive regulators of osteoclastogenesis (Garlet et al., 2006). Interestingly, in this set-up this did not result in higher numbers of osteoclasts, but rather lower numbers. However, Cao et al. showed that TNF-α pre-treatment prevented the differentiation of TRAcP+ osteoclasts generated from one type of mouse osteoclast precursors on plastic, in contrast to those seeded on bone (Cao et al., 2017). Naruishi et al. suggested that although TNF-α and IL-1β are both pro-inflammatory cytokines, the synergy of TNF-α and IL-1β could inhibit inflammatory responses in the acute phase (Naruishi, 2022). Bloemen et al. found that after three days of direct co-cultures of periodontal ligament fibroblasts with PBMC, *IL-1β* expression increased significantly compared to monocultures (Bloemen et al., 2010). Our data suggest that a similar process is seen without the direct contact of cells. In our case study, the increased expression of inflammatory genes by PBMC indirectly co-cultured with GF pre-treated with cytokines could indicate that the PBMC have changed phenotype due to the inflammatory stimulus of the GF, resulting in a crosstalk that amplifies the expression of inflammatory cytokines by GF. This is consistent with a shift in cell type, as suggested in the study of Sprangers et al. (Sprangers et al., 2016a). They proposed that, after treatment with IL-17 A, a pro-inflammatory cytokine, one of the monocyte subsets from human peripheral blood commits to becoming a mature cell type different from the bone-resorbing osteoclast lineage. The contribution of monocytes to populations of mature immune cells is dramatically increased in various inflammatory conditions (Sprangers et al., 2016a; Sprangers et al., 2016b).

*IL-4* expression by PBMC was increased under inflammatory conditions. It is known to inhibit the stimulation of osteoclast and the presence of IL-4-producing cells is significantly higher in periodontal lesions than in gingival tissues (Ujiie et al., 2016). Ujiie et al. demonstrated that IL-4 present in GF-conditioned medium inhibits osteoclast differentiation (Ujiie et al., 2016). In our study, *IL-4* expression was mainly detected in PBMC. Interestingly, GF supernatant, when cultured under inflammatory conditions, stimulated *IL-4* expression by PBMC. IL-4 is critical for the skewing of macrophages toward an M2 phenotype (Celik et al., n.d.), suggesting that some of the monocytes became macrophages. This may also explain why this experimental condition resulted in fewer osteoclasts.

Exposure of GF to pro-inflammatory cytokines increased their gene expression of *IL-6*. Sawada et al. demonstrated a relationship between IL-1β and IL-6, showing that IL-1β induced IL-6 secretion in GF (Sawada et al., 2013). IL1β might up-regulate functionally active IL-6 through an autocrine loop. Possibly the high levels of *IL-6* gene expression reflect a similar additive effect of IL-1β exposure on *IL-6* expression in our indirect co-culture system.

Furthermore, IL-10 concentration increased significantly in GF supernatants pre-treated with inflammatory cytokines. It is a cytokine known for its anti-inflammatory properties, although it is thought to have a pleiotropic role (Morandini et al., 2011; Mocellin et al., 2003). It negatively affects the early stage of differentiation of osteoclast progenitors into pre-osteoclasts (Ujiie et al., 2016). Indeed, IL-10 presents a protective role toward bone tissue destruction, inhibiting the RANKL-RANK system (Morandini et al., 2011). In our study, IL-10 may play a role in reducing osteoclast formation in inflammatory conditions.

MCP-1 has been widely accepted as a profound inflammatory mediator, having both pro-inflammatory and anti-inflammatory roles (Brylka and Schinke, 2019; McDonald et al., 2021; Mulholland et al., 2019). Our results showed that MCP-1 concentration was higher in the supernatant of GF without inflammation in indirect co-culture. Interestingly, *MCP-1* gene expression by PBMC increased when they were indirectly co-cultured with pre-treated GF. GF pre-treated with cytokines could therefore exert an anti-inflammatory effect on PBMC. These results suggest that MCP-1 is one of the soluble factors via which GF and osteoclast precursors communicate in a paracrine manner.

However, as mentioned previously, a single PBMC donor was used in this case study. This is one of the limitations of the study as it could be argued that, without repetition with other PBMC donors, it remains unknown whether the results will prove generalizable. However, we are aware that in the present set-up, the variations come from the five different GF donors, and not from the possible variation introduced by extra PBMC donors. This approach was chosen as the aim of this study is to learn more about paracrine signaling by GF. Further studies will have to confirm the results, using both multiple GF donors and multiple PBMC donors.

In conclusion, GF appear to communicate with osteoclast precursors via soluble factors. In this case study, they were able to stimulate formation of osteoclasts, especially in the absence of direct cell-cell contact. Conversely, PBMC also influenced GF gene expression when they were not in direct contact. During inflammation, we observed a strong back and forth communication between GF and PBMC. Interestingly, incubation of GF with a cocktail of inflammatory cytokines for 24 h reduced rather than enhanced their stimulation of osteoclast formation. This could be explained by an increase in *IL-4*, which directs the differentiation of monocytes into macrophages, and IL-10, and a decrease in MCP-1. Whether this is due to the duration of exposure is yet to be determined, but the present results suggest that GF that sense inflammation may also down-regulate anabolic responses such as bone degradation.

## Funding

This research was funded by the University Hospital of Rennes, France (CHU Rennes, France).

## CRediT authorship contribution statement

**Solen Novello:** Writing – review & editing, Writing – original draft, Visualization, Investigation, Funding acquisition, Formal analysis, Data curation, Conceptualization. **Ton Schoenmaker:** Writing – review & editing, Validation, Supervision, Methodology, Investigation, Formal analysis, Data curation, Conceptualization. **Teun J. de Vries:** Writing – review & editing, Validation, Supervision, Methodology, Investigation, Conceptualization. **Behrouz Zandieh Doulabi:** Writing – review & editing, Methodology, Investigation. **Astrid D. Bakker:** Writing – review & editing, Supervision, Project administration, Methodology, Conceptualization. **Marja L. Laine:** Writing – review & editing, Supervision, Project administration, Conceptualization. **Ineke D.C. Jansen:** Writing – review & editing, Validation, Supervision, Methodology, Conceptualization.

## Declaration of competing interest

The authors declare that they have no known competing financial interests or personal relationships that could have appeared to influence the work reported in this paper.

## Data Availability

Data will be made available on request.
